# Supplementary figures and images for: Experience-dependent evolution of odor mixture representations in piriform cortex
Source: PLoS Biol. 2023 Apr 25;21(4):e3002086. doi: 10.1371/journal.pbio.3002086 (PMC10129003; doi:10.1371/journal.pbio.3002086)

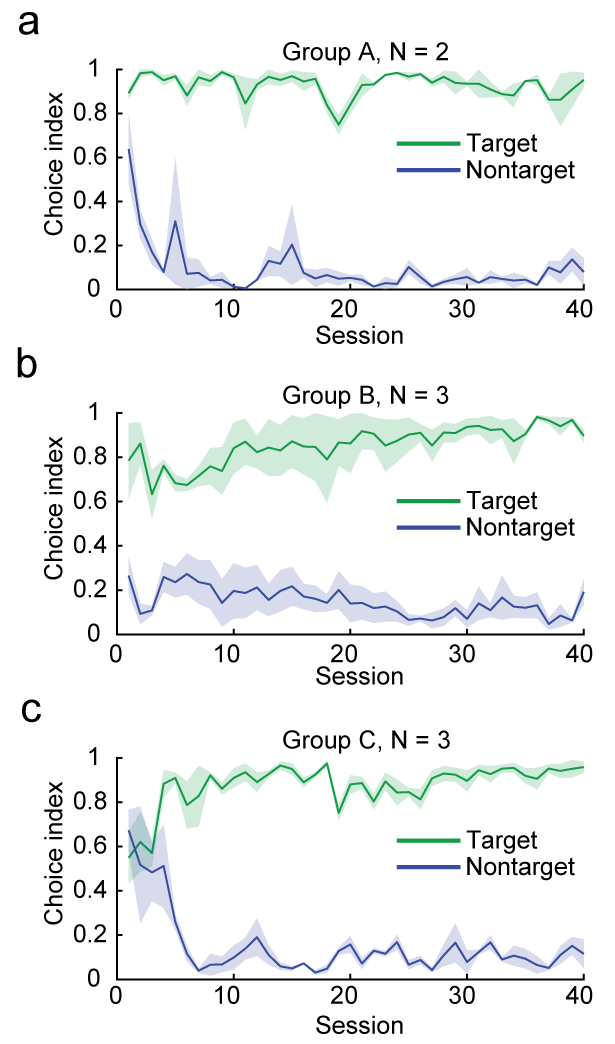

Supplement: S1 Fig — Average choice index across the first 40 sessions for groups of mice trained on different target mixtures. Session 1 for each mouse is the first session with blocked trials. (a) Mouse group A trained on target mixture: Heptanal, Propyl Acetate, Isoamyl acetate. (b) Mouse group B trained on target mixture: Allyl buterate, Ethyl valerate, Methyl tiglate. (c) Mouse group C trained on target mixture: Ethyl tiglate, Allyl tiglate, Methyl tiglate. Shading represents mean +/− SEM. The underlying data for this figure are available for download from https://datadryad.org/stash/share/bC3NdXWDllJZYrRtq60q0WDQYhjZZuLulv91dm9WcYU. (TIF) [file pbio.3002086.s001.tif]

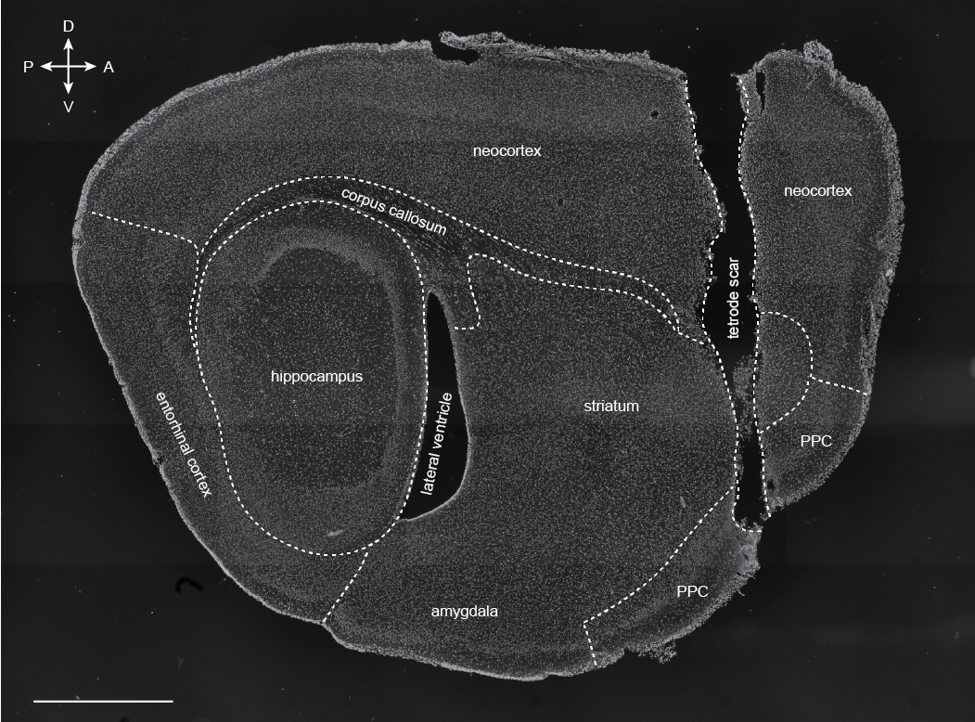

Supplement: S2 Fig — This sagittal section comes from the right hemisphere of mouse S. The oval shape of the hippocampus, the shape and position of the lateral ventricle, as well as the absence of lateral olfactory tract confirm that the tetrodes were placed in the PPC and not the APC. A: anterior. P: Posterior. D: dorsal. V: ventral. Scale bar: 1 mm. DAPI staining. We did not trace the border between striatum and amygdala as we could not confidently determine it. (TIF) [file pbio.3002086.s002.tif]

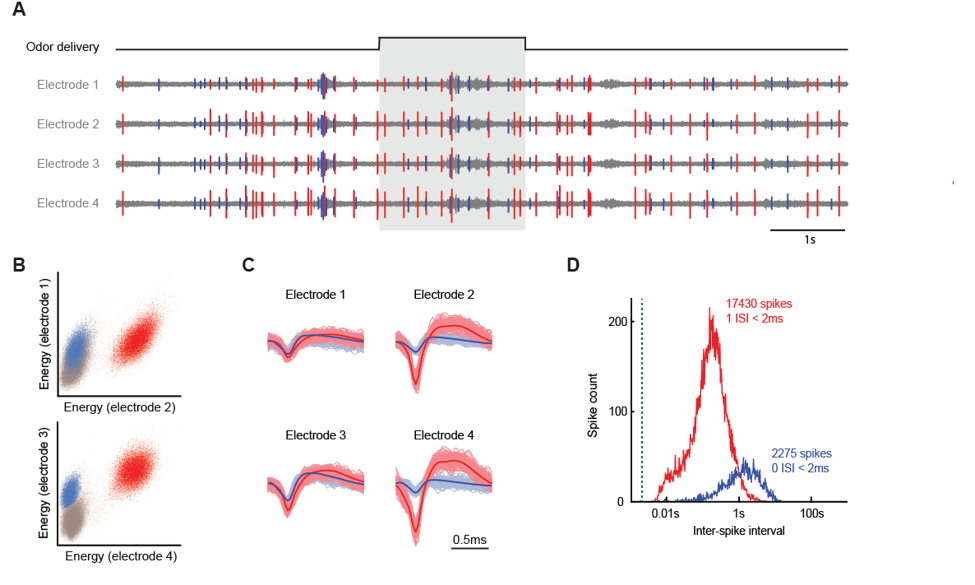

Supplement: S3 Fig — (a) Example of tetrode recoding. The 4 electrodes are from the same tetrode. Two single units are shown in red and blue. Grey: odor delivery. Example from mouse E, session 2-22-2017, tetrode #4. (b) Single unit clustering. The red and blue units are the same as (a). (c) Clustered single units. The red and blue units are the same as (a). The lighter traces show the 100 first extracellular action potential of each unit superimposed. The darker traces show the average extracellular action potential. (d) Single unit confirmation. The red and blue traces belong to the red and blue units from (a). The interspike interval analysis reveals the presence of a refractory period. The underlying data for this figure are available for download from https://datadryad.org/stash/share/bC3NdXWDllJZYrRtq60q0WDQYhjZZuLulv91dm9WcYU. (TIF) [file pbio.3002086.s003.tif]

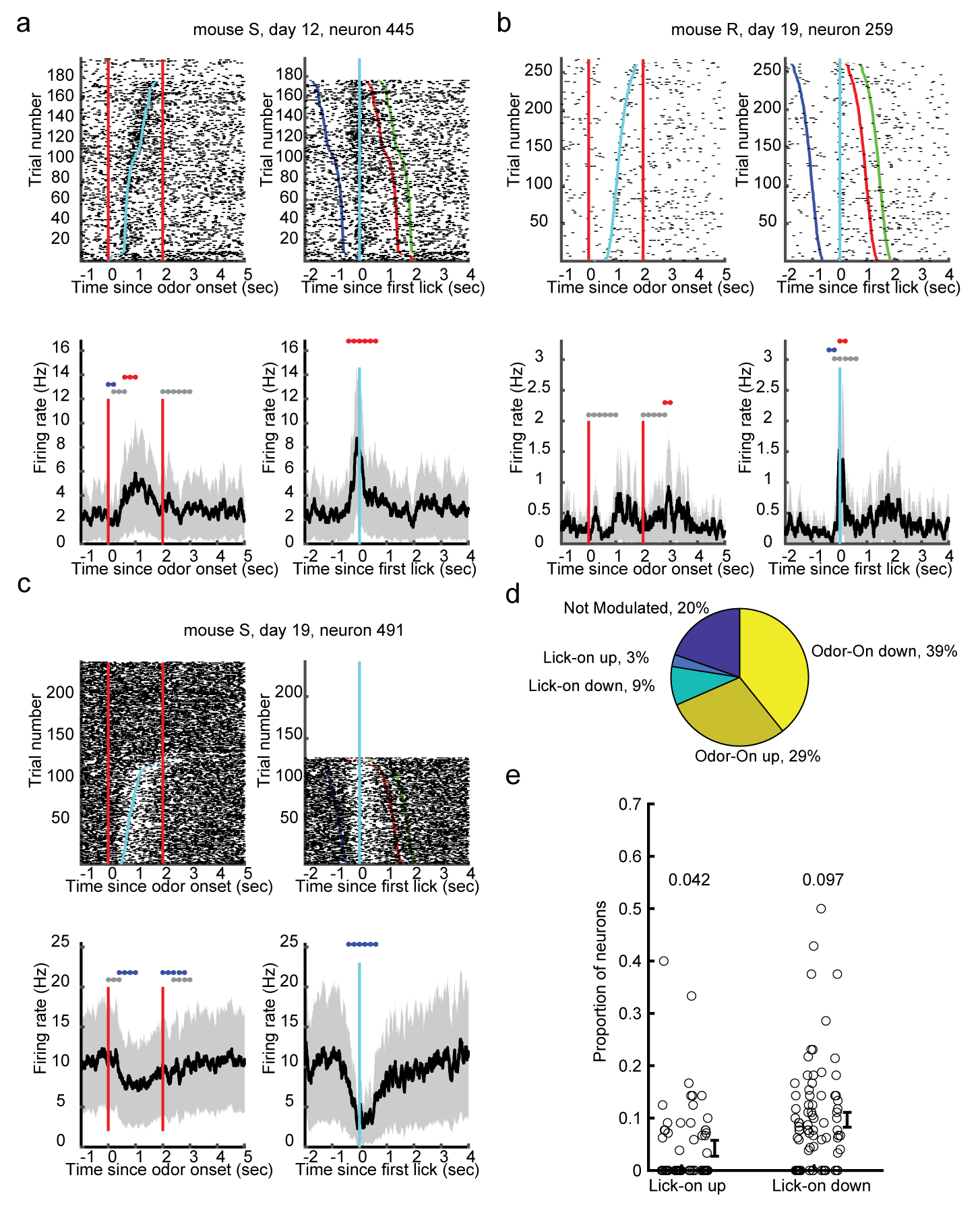

Supplement: S4 Fig — (a-c) Three example neurons that are modulated at first lick or reward. Raster plots (top) and PSTHs (bottom) of odor-aligned (left) or lick-aligned (right) trials. Red lines depict odor onset and offset; blue dots depict lick times. Green dots represent the end of the response period. (d) Proportion of the total number of neurons recorded with significant task-relevant responses. (e) Proportion of neurons in each session (circles) with lick-on up and lick-on down responses. Average proportions are above each group. Error bars and shading represent mean +/− SEM. The underlying data for this figure are available for download from https://datadryad.org/stash/share/bC3NdXWDllJZYrRtq60q0WDQYhjZZuLulv91dm9WcYU. (TIF) [file pbio.3002086.s004.tif]

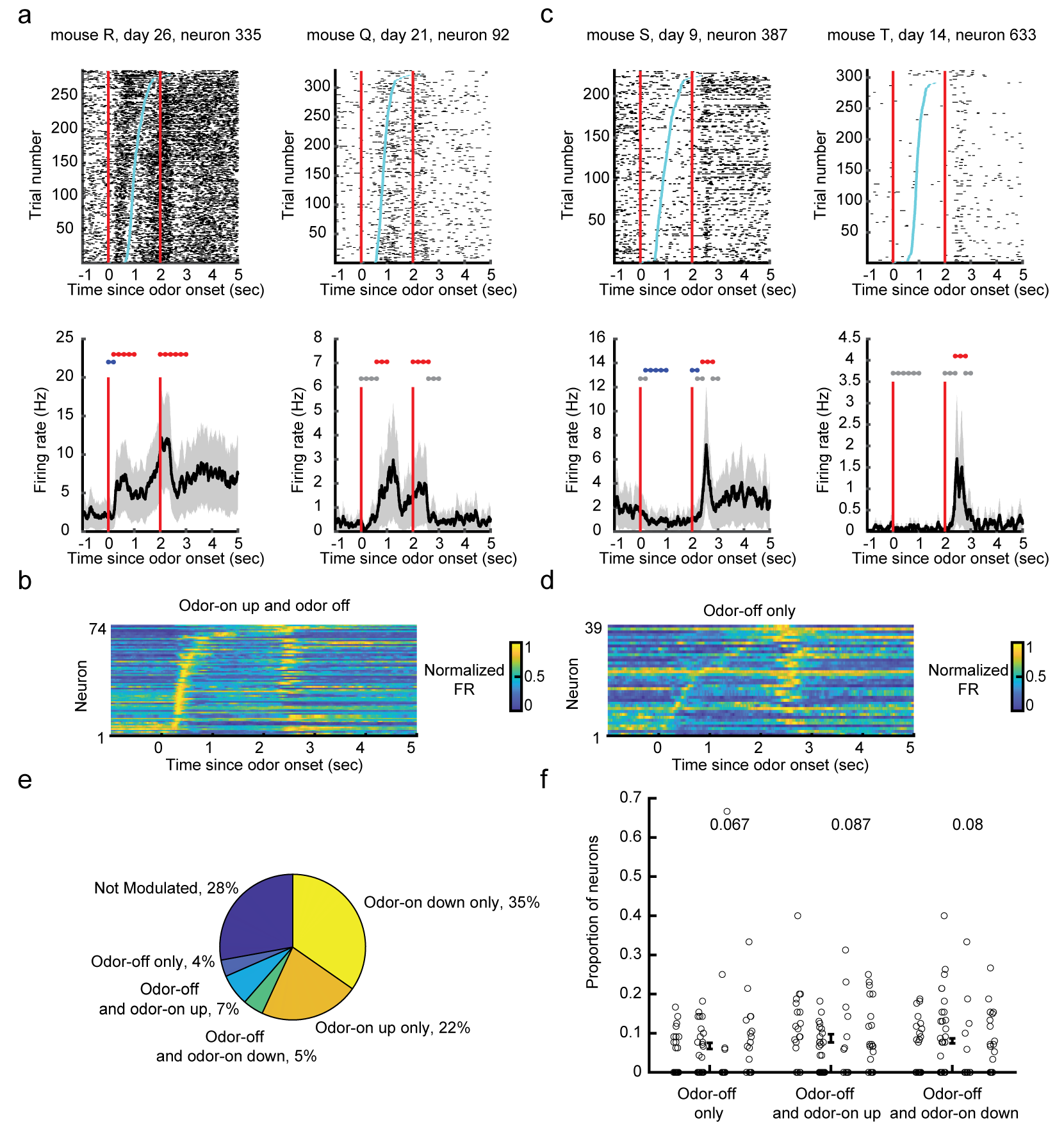

Supplement: S5 Fig — (a) Two example neurons (left and right) that have both a significant odor-on up and odor-off response. Raster plots (top) and PSTHs (bottom) of odor aligned trials. Red lines depict odor onset and offset; blue dots depict lick times. (b) The normalized firing rate of all odor-on up and odor-off responsive neurons. (c, d) Same as in a and b but with odor-off only neurons. (e) Proportion of the total number of neurons recorded with significant task-relevant responses. (f) Proportion of neurons in each session (circles) with different odor-off responses. Average proportions are above each group. Error bars and shading represent mean +/− SEM. The underlying data for this figure are available for download from https://datadryad.org/stash/share/bC3NdXWDllJZYrRtq60q0WDQYhjZZuLulv91dm9WcYU. (TIF) [file pbio.3002086.s005.tif]

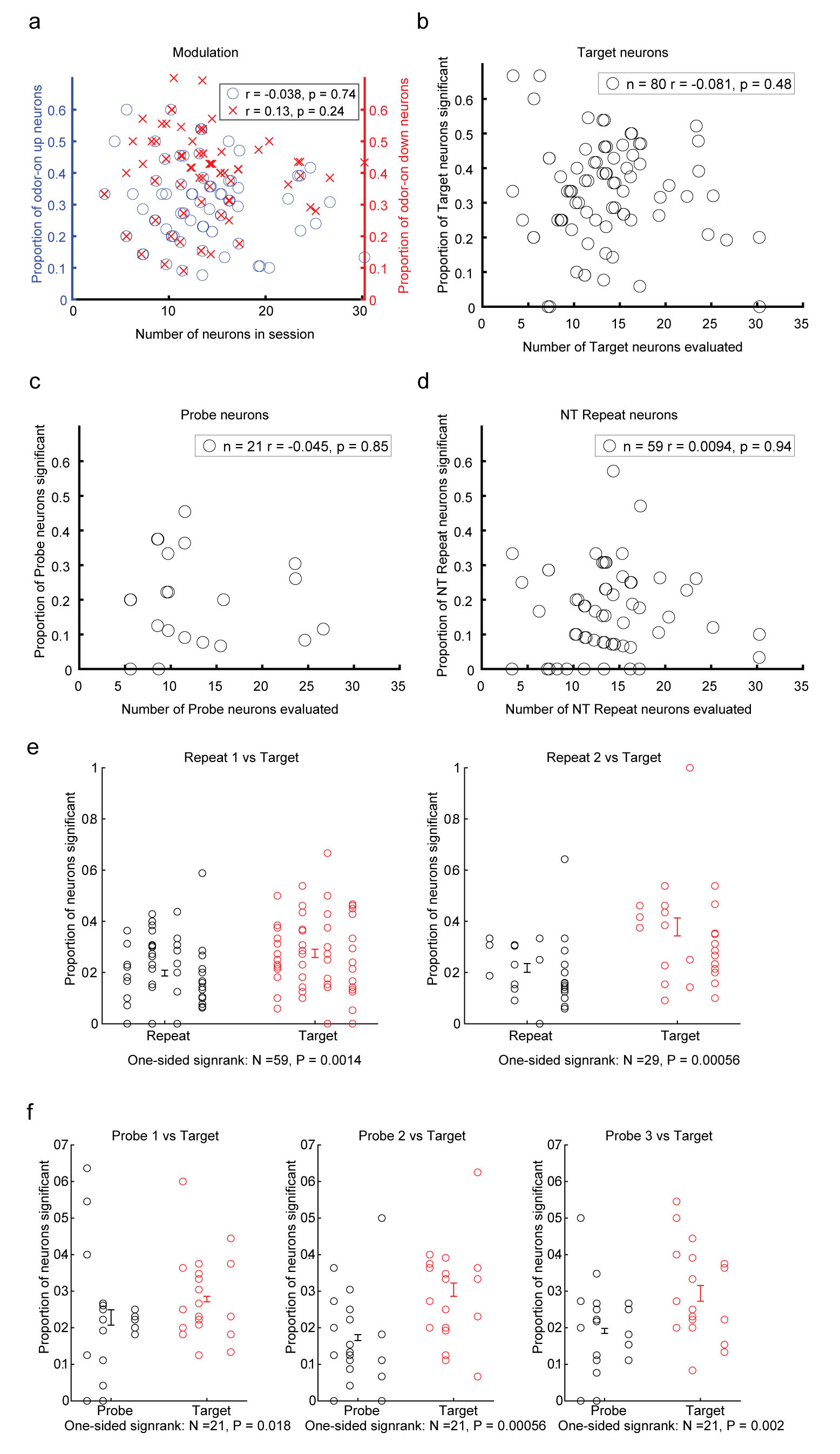

Supplement: S6 Fig — (a) The number of neurons recorded simultaneously in each session compared to the proportion of modulated neurons (odor-on up on the left axis, odor-on down on the right axis, Pearson’s correlation). (b) The number of neurons evaluated for each session compared to the proportion of target-selective neurons (Pearson’s correlation). (c) The number of neurons evaluated for each session compared to the proportion of probe-selective neurons (Pearson’s correlation). (d) The number of neurons evaluated for each session compared to the proportion of nontarget repeat-selective neurons (Pearson’s correlation). (e, f) We repeated comparisons while down-sampling the number of trials to control for the uneven number of trials across trial types. Details of this procedure are in the Trial down-sampled comparisons section of the methods. (e) Proportion of neurons significantly selective for NT repeat (black) and target (red) trials. One-sided signed rank test to determine whether there is a higher proportion of target-selective neurons than NT repeat-selective neurons separately for each of the 2 possible repeats within a session (left vs. right). (f) Proportion of neurons significantly selective for probe (black) and target (red) trials. One-sided signed rank test to determine whether there is a higher proportion of target-selective neurons than probe-selective neurons separately for each of the 2 possible repeats within a session (left vs. right). See methods for more details. Error bars represent mean +/− SEM. The underlying data for this figure are available for download from https://datadryad.org/stash/share/bC3NdXWDllJZYrRtq60q0WDQYhjZZuLulv91dm9WcYU. (TIF) [file pbio.3002086.s006.tif]

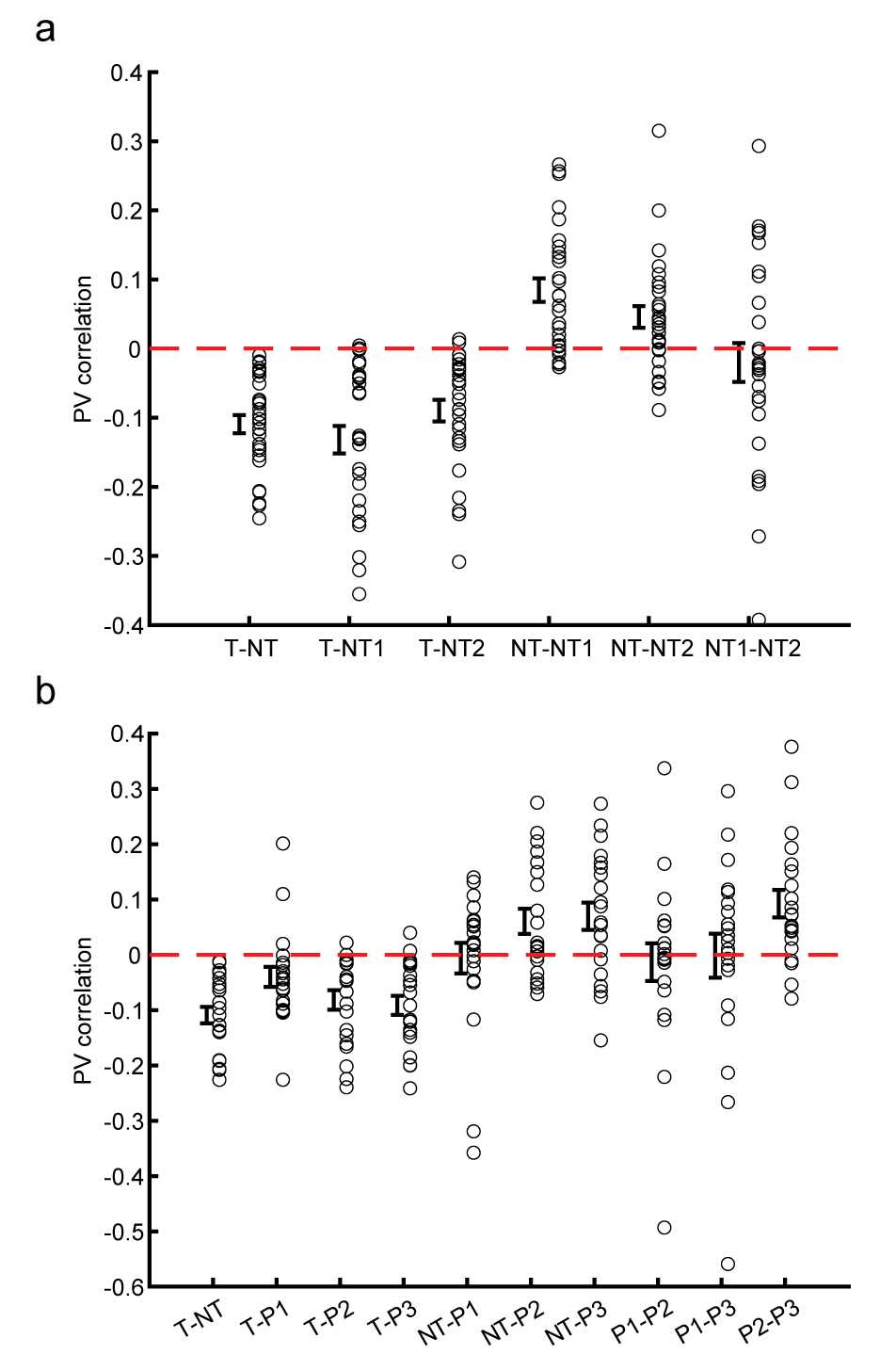

Supplement: S7 Fig — Average population vector corrections for each session (open circles) between each trial type category in (a) overlearning sessions with at least 2 nontarget repeats, and (b) probe sessions. T–Target, NT–Nontarget, NT1-2 –Nontarget repeats 1–2, P1-3 –probe 1–3. Error bars represent mean +/− SEM. The underlying data for this figure are available for download from https://datadryad.org/stash/share/bC3NdXWDllJZYrRtq60q0WDQYhjZZuLulv91dm9WcYU. (TIF) [file pbio.3002086.s007.tif]

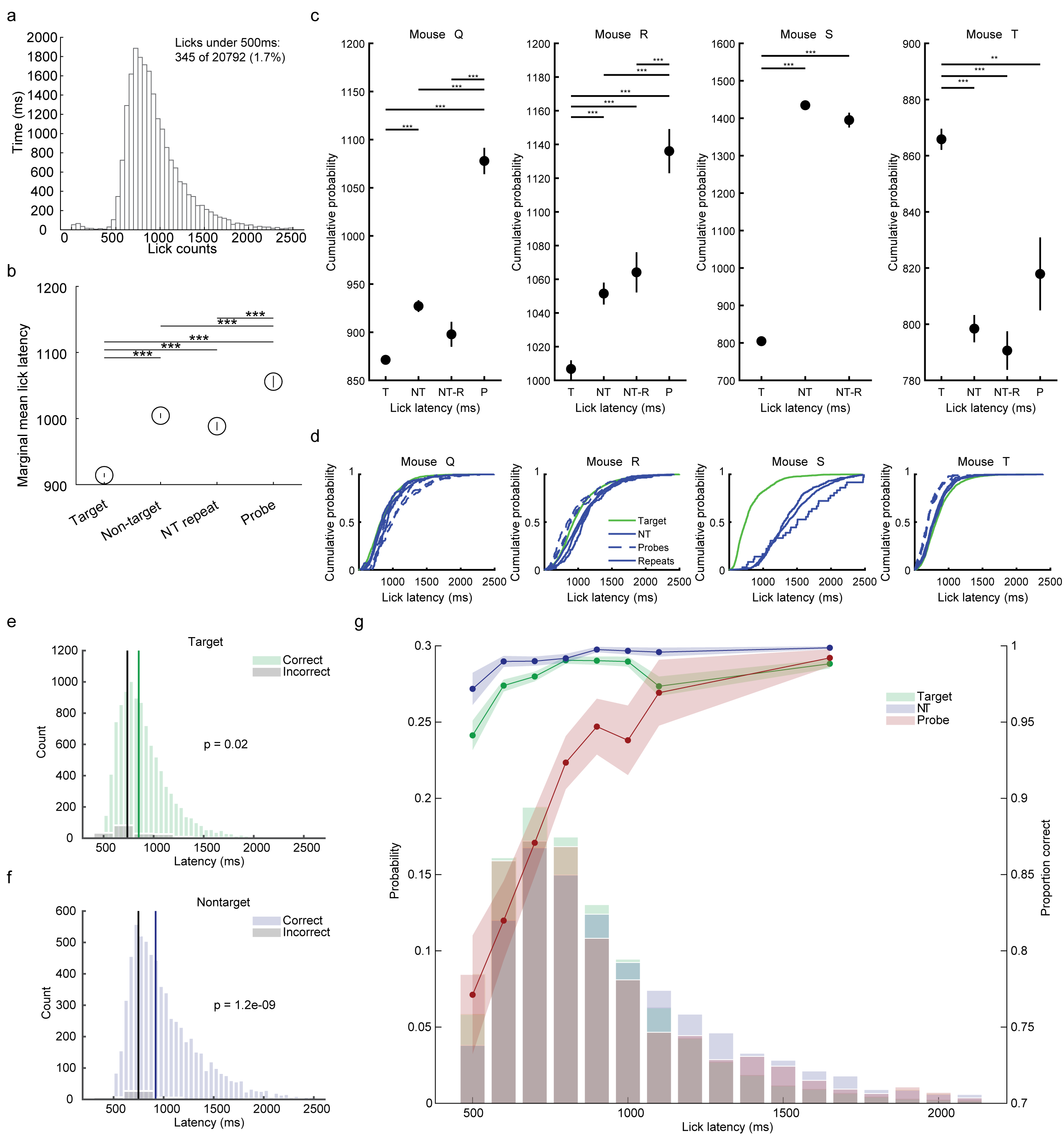

Supplement: S8 Fig — (a) Distribution of all lick latencies. In subsequent analyses, licks occurring before 500 ms (1.7% of all licks) were excluded. (b) Trial-type lick latency differences. Marginal mean lick latency by trial type from a 3-way ANOVA with trial type, mouse, and session. Main effect of trial type: F(20288,3) = 176.3, P = 7.2 × 10−133. Main effect of mouse F(20288,3) = 853.9, P = 0. Main effect of session F(20288,3) = 973.2, P = 9.6 × 10−209. Post hoc Bonferroni tests between trial types shown with asterisks. (c) Marginal mean lick latency for each mouse by trial type from a 2-way ANOVA with trial type and session. All mice showed a main effect of trial type (P < 1 × 10^−20^ in all mice). Post hoc Bonferroni tests between trial types shown with asterisks. * P < 0.05, ** P < 0.01, ** P < 0.001. Error bars in a represent mean +/− S.E. Note different y-axis scales. (d) Lick latency distributions for all trials of target, nontarget, nontarget repeats, and probe trials, for each of the 4 mice. (e) Lick latencies for all target trials and (f) nontarget trials, separately for correct and incorrect trials. Only trials during probe sessions were used. (g) Left axis: A histogram of lick latencies during probe sessions by trial type. 100 ms bins. Right axis: The proportion of correct trials as a function of lick latency. Only probe sessions were used. The last data point includes latencies of 1,200–2,200 ms. Shading represents mean +/− SEM. In panels b-g, we only considered trials in which the first lick came more than 500 ms after odor onset. The underlying data for this figure are available for download from https://datadryad.org/stash/share/bC3NdXWDllJZYrRtq60q0WDQYhjZZuLulv91dm9WcYU. (TIF) [file pbio.3002086.s008.tif]

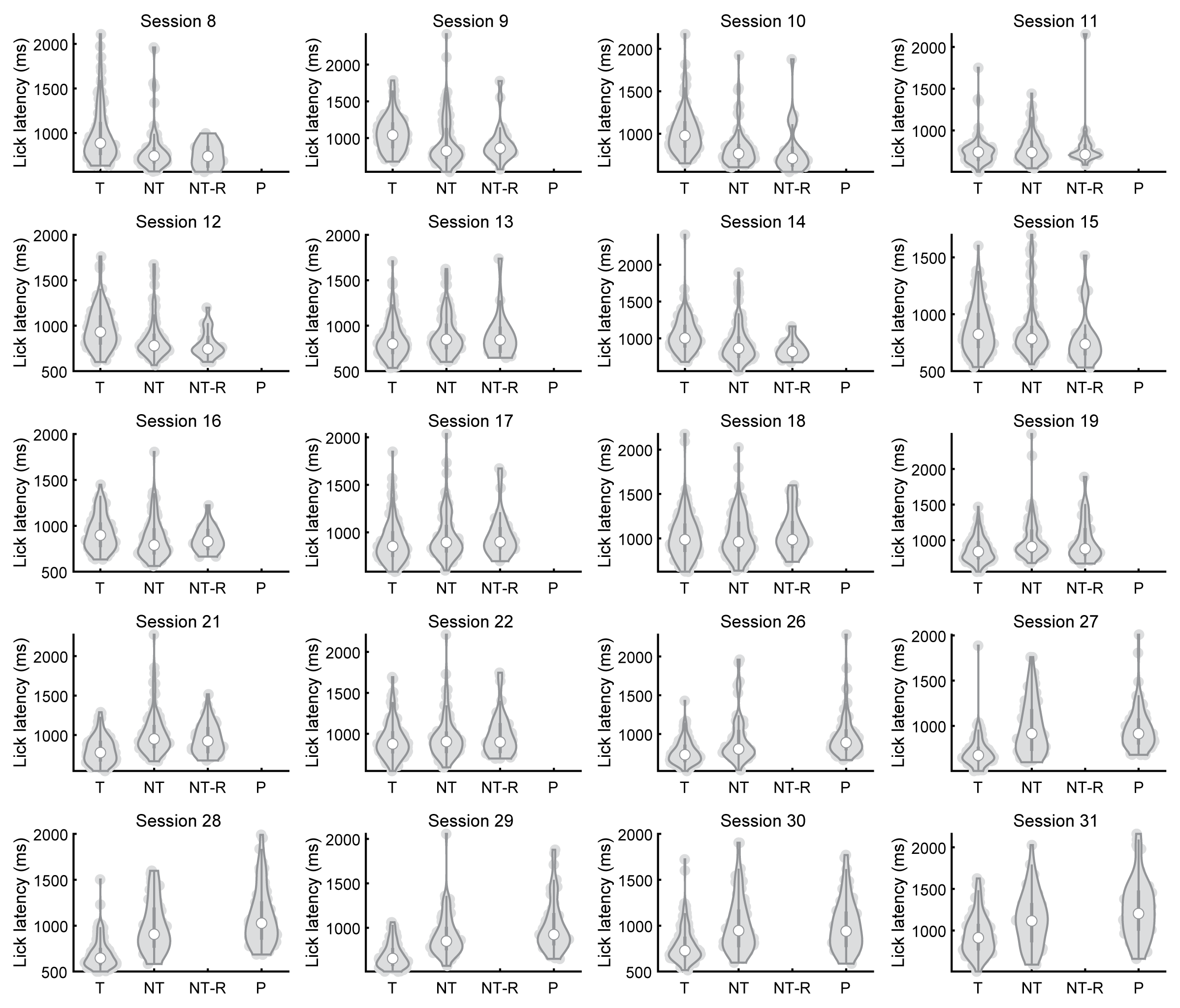

Supplement: S9 Fig — Violin plots of mouse Q’s lick latencies in each session. X-ticks denote trial types. T: target; NT: nontarget; NT-R: nontarget repeat; P: probe. The underlying data for this figure are available for download from https://datadryad.org/stash/share/bC3NdXWDllJZYrRtq60q0WDQYhjZZuLulv91dm9WcYU. (TIF) [file pbio.3002086.s009.tif]
