# Supplementary material for: Experience-dependent evolution of odor mixture representations in piriform cortex
Source: PLoS Biol. 2023 Apr 25;21(4):e3002086. doi: 10.1371/journal.pbio.3002086 (PMC10129003; doi:10.1371/journal.pbio.3002086)
Supplement: Attachment — Submitted filename: TargetPaper_Response_to_Reviewers_Draft4.pdf [file pbio.3002086.s010.pdf]

We thank the reviewers for their comments on our manuscript. Below are our responses to each reviewer in turn. The reviewer's comments are reproduced in non-bold **font, with our responses to each point in bold font.**

Reviewer #1: In this manuscript, the authors describe odor responses of neurons in the posterior piriform cortex and how these responses evolve when mice learn to recognize a specific combination of odors from other combinations. They find that more neurons respond to the "target" combination than other odor combinations. They also find that, with over-training, the response statistics continue to change despite no apparent change in the behavioral output. The authors find that the discriminability of target response patterns relates to the mice's ability to distinguish the specific odor combination from related but different odor combinations, suggesting a potential benefit of covert learning with over-training.

The posterior piriform cortex is still relatively poorly explored and understood. The current study, therefore, adds a significant contribution. The behavioral paradigm is clever; overall, the experiments are designed well, and the data are of high quality. The manuscript may, however, benefit from greater clarity in presentation and description.

(1) It is currently somewhat ambiguous if this study is about recognizing a specific odor combination/association with a reward (on one side) or if this is a consequence of repeated exposures. For example, previous studies on the anterior piriform cortex suggest that repeated encounters or exposure can affect the olfactory responses in this region (Schoonover et al., DOI: https://doi.org/10.1038/s41586-021-03628-7). In this study, the target odor combination is the most frequently encountered mixture, more than the non-target repeat odor combinations. To truly distinguish "relevance" vs. "exposure", may require a dissociation of the odor and the reward while keeping stimuli the same (e.g., Adefuin et al., DOI: https://doi.org/10.7554/eLife.76882). Although such an experiment is beyond the scope of this study, some clarification in the discussion may be beneficial.

We thank Reviewer 1 for their careful assessment and insightful comments on our manuscript. We agree with the reviewer that because of the task design we are unable to separate experience from learning, although both are forces at play. We hoped we were being clear with about the shortcomings of this aspect. In the first section of the Discussion we stated:

"The largest over-representation was of the target odor mixture, likely showing the effect of both the repeated exposure of the odor-mixture and the reinforced dimension of the task"

However, we agree with the reviewer that an expanded discussion is warranted.

Schoonover et al. mainly focus on differences in the stability of representation (drift) with exposure (which we cannot evaluate, since we do not track the same neurons across days, but only take snapshots of the population activity each day), and not over-representation. There is, however, other evidence of over-representation with exposure. As suggested, we have added a paragraph to the discussion as explanation of the shortcomings and context of this study, re-printed here:

"Our data also does not speak to how much of the over-representation of target-selective neurons occurs from repeated experience or from learning. We do not have neural data

from early learning to see how this over-representation emerges, and the target condition is the most frequent as well as the most reinforced. Both experience (Pashkovski et al., 2020) and reinforcement (Calu et al., 2007) have led to over-representation of selective neurons within the piriform cortex, and we believe both forces are at play here. Schoonover and colleagues also found that experience alone can also reduce drift of the aPC neural representation over time (Schoonover et al., 2021). Exploration of that effect cannot be assessed here, as we did not follow the same neurons over days. It is likely that the active component and the go-go structure of our task both contribute to differences in pPC representation along with the mere exposure of odors (Wang et al., 2019), and future work exploring how each of these task demands change separately across time would be fruitful."

We have also changed the title of the paper to "*Experience-dependent*" from "learning-dependent" so as not to mislead the reader.

(2) In the plot presented in Figure 2b, the statistics reported show a wide distribution. Does this relate to the sample size? It may help if the number of units encountered per session is given somewhere.

We agree with the reviewer that there is a wide distribution and thank the reviewer for their hypothesis. We did not find that the sample size on a given session is related to the proportion of modulated neurons. We have added panels to Supplemental Figure 6 (panel A) to show this (re-printed below).

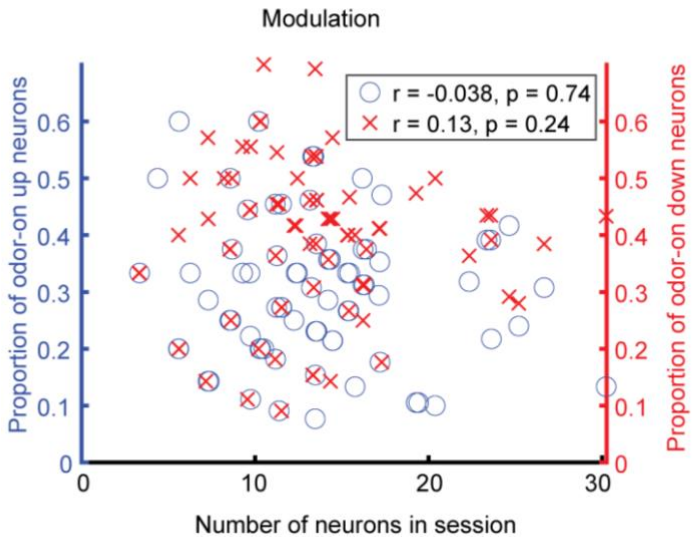

We have also added a statement to the main text that states:

"The proportion of neurons with significantly modulated firing rates is not related to the number of neurons recorded in any given session (Supplementary Figure 6; Range of 3-30 neurons per session, mean = 12.2, median=13)."

We also added the number of units per session in the same section (see above) as well as for those of analyses in Figure 4, as Reviewer 3 had a similar suggestion regarding those sessions.

We hypothesize that the variability relates to the typical experimental variability of recording a small sample of a brain area. If we were able to consistently record from a higher proportion of the piriform neurons at once from each animal, ideally all of them, then we would expect less variability. This variability could come from subsampling slightly different layers, genetically defined neurons, anatomical locations etc.

(3) In calculating the correlation between population vectors, the authors seem to have normalized each unit's firing rates, normalized the correlation coefficients, and expressed them as z-scores. This is somewhat confusing and makes it harder to compare with other studies (e.g., comparison of correlation levels observed in the anterior piriform cortex e.g., Bolding and Franks, DOI: https://doi.org/10.7554/eLife.22630). Could the authors either use a simpler correlation coefficient or, alternatively, explain why their method was chosen.

We thank the reviewer for their consideration of differences between other studies and for the opportunity to explain and justify our methods more thoroughly.

We z-scored the neuron's firing rates in order to normalize the variance across the population, "such that each neuron contributed equally to subsequent analysis" (Methods, Population Analysis, last sentence). There are other aspects to this normalization that are appealing as well. For instance, since we are using the post-odor period, which for negatively modulated neurons especially, have many trials without spikes during this window. Therefore, there could be a trial where no neurons have any spikes, however this can mean something different for each neuron (based on their firing in other trials). Because there is no variance in this trial, there would not be a way to calculate a correlation with this trial.

We z-scored the population vector correlations in order to more accurately compare changes across trial types and time. By normalizing the comparisons within a session we reduce spurious variability that can come from different numbers of neurons being recorded in each session (the spread/variability of PV correlations will be larger when there are fewer neurons, and smaller with more neurons if they aren't normalized). Spurred by the reviewer's comment, we replicated the analyses without normalizing the population vectors and below show that figures 7 and s7 are similar to those in the main text:

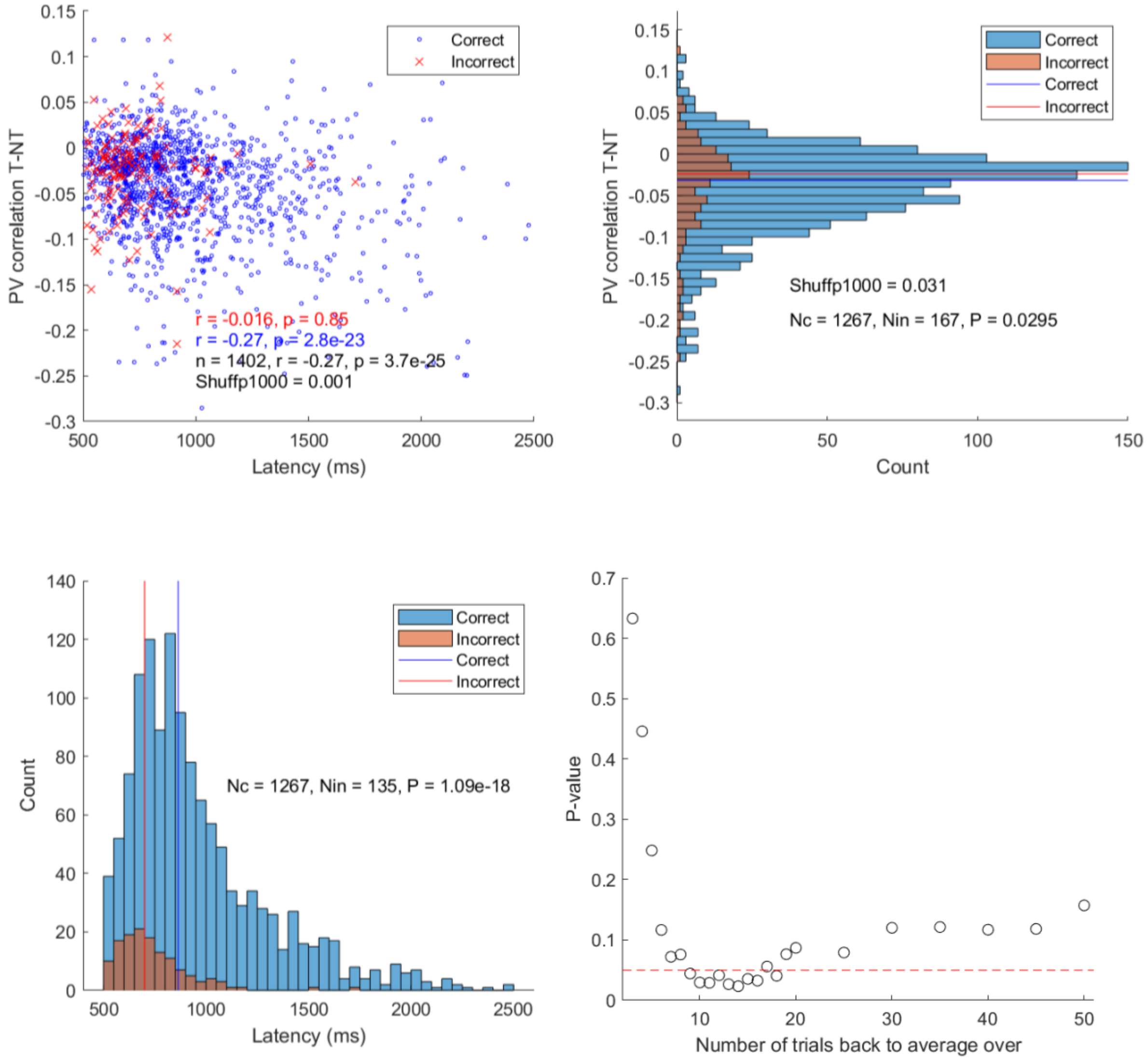
PV correlation T-NT
Latency (ms)
Correct
Incorrect
r = -0.016, p = 0.85
r = -0.27, p = 2.8e-23
n = 1402, r = -0.27, p = 3.7e-25
Shuffp1000 = 0.001
PV correlation T-NT
Count
Correct
Incorrect
Correct
Incorrect
Shuffp1000 = 0.031
Nc = 1267, Nin = 167, P = 0.0295
Count
Latency (ms)
Correct
Incorrect
Correct
Incorrect
Nc = 1267, Nin = 135, P = 1.09e-18
P-value
Number of trials back to average over

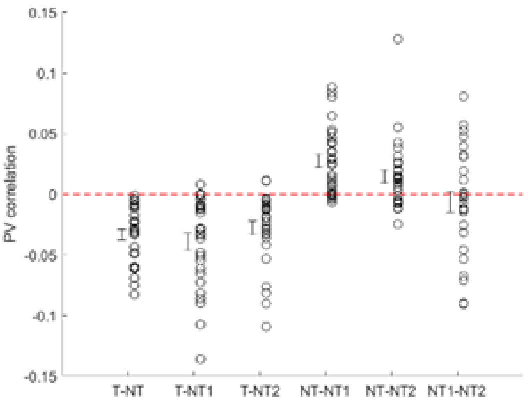

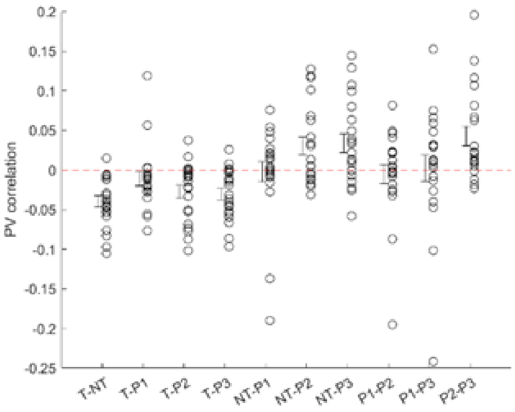

We added justification of the population vector normalization in the "Population vector corrections" section of the Methods and an explanation of the replicated effects shown above to the same section, re-printed here:

"We z-scored the population vector correlations in order to more accurately compare changes across trial types and time. By normalizing the comparisons within a session, we reduce spurious variability that can come from different numbers of neurons being recorded in each session (the variability of population vector correlations will be larger when there are fewer neurons, and smaller with more neurons if they are not normalized). We also replicated analyses in Figure 7 without normalizing the population vectors and found similar and significant results."

(4) How does the proportion of responsive neurons relate to the correlation coefficient? Clarifying this may help relate the analysis presented in Figure 7 to the covert learning described in Fig. 5 d/e.

As we may expect, due to the fact that the proportion of target-selective neurons increase across sessions and the correlation coefficients between target and nontarget trials decreases (target-nontarget discriminability increases) across the same sessions, we see a negative relationship between those two factors.

We hoped to make the logic of moving from the single-neuron analyses to population analyses clear by stating in the Results: "To assess the possible consequences of this increase in the proportion of task-selective neurons, we performed categorical (target vs. everything else) decoding using the population of simultaneously recorded piriform neurons on each day." We hope we understood the intent of the reviewer in this question, but if the reviewer was hoping for more or different clarification we are open and willing to investigating and including additional information.

(5) The statement in the introduction, "... but in that case they have access to all relevant odors and do not have to depend on memory." (page 3) is unclear. Since this seems vital to explaining this study's goal, a more straightforward statement may help.

We thank the reviewer for their suggestion and we agree that the wording was not clear. We have changed that sentence (re-printed below) and hope that the distinction we are

making is now apparent.

However, in a rodent's natural environment, odors can be more complex and there may be hundreds of overlapping odors that need to be distinguished from a goal or target odor. Mice can detect the presence of a single odor within a large odor mixture (17) in a go/no-go design. To investigate how pPC representations change with experience, we developed a complex odor task where mice learned to categorize one target odor mixture as different from all the hundreds of other odor mixtures.

(6) The method describes the electrode placement at 3.8 mm lateral to the Bregma. All recordings presumably occurred ipsilateral to the reward port associated with the target odor combination. It may help to spell this out, even if the relationship to the tongue movement is irrelevant.

We agree and have added this statement to the Surgery section of the Methods:

(All recordings occurred ipsilateral to the reward port associated with the target odor combination.)

(7) Typo: 200 mm should probably read 200 um (page 26).
(8) Typo: "performed a" appears twice on page 35.

We thank the reviewer for their careful read; we have corrected both of these typos.

Reviewer #2: In this manuscript, Berners-Lee, Shtrahman and colleagues address two important yet poorly understood questions in olfaction: how odor mixtures are represented in the olfactory cortex, and how the representation of odors in piriform is modulated by learning and experience.
The authors trained head-fixed mice to obtain a water reward at one lick port in response to a specific target odor mixture, and at the other lick port in response to a large panel of non-target odor mixtures. Extending this basic odor discrimination task after initial learning, experiments were also performed on overtrained mice and including non-target repeats and probe mixture trials. Tetrode recordings in the posterior piriform cortex revealed target odor mixture-specific tuning properties. Furthermore, overtraining resulted in improved odor coding and better task performance on difficult to categorize odor mixtures.
Taken together, the authors here develop a novel odor mixture discrimination task in mice, provide a detailed description of odor mixture representations in the piriform cortex, and identify experience-dependent changes in odor representations that are consistent with improved behavioral performance. Before publication, the authors should clarify the task design and provide additional analyses to support their main findings.

Major points:

1) The description of the task design is confusing. For example, I suspect that basic non-target odor mixtures contain 3 components - this is not specified, and Figure 1a appears to suggest that non-target odor mixtures contain many components. How do non-target repeats differ from targets? If repeated, how do the authors distinguish between mice discriminating targets from non-targets versus mice learning about non-targets? Figure 1b appears to suggest that target and non-target trials are blocked, the methods section suggests that they are not.
The task design is central to the entire study and its description should be clear and unambiguous for a general, non-expert audience.

We thank Reviewer 2 for their careful assessment and insightful comments on our manuscript.

We agree with the reviewer that we did not make it clear that three nontarget odors comprise one nontarget odor mixture. To remedy this, we edited the figure legend of Figure 1:

"(a) Odor panel. Three odor components make up the target mixture (top) and the nontarget odors are drawn from 13 other components (bottom). Each nontarget trial consists of 3 of these 13 components."

We also added a clarifying sentence to the first paragraph of the Results, re-printed below:

"The nontarget odor mixtures also contained three single odor components randomly drawn from thirteen odors."

In order to clarify the interleaved trial structure, we added explanation to the figure legend of Figure 1:

“(b) Behavioral set up (top) and stimulus structure (bottom). Colored boxes mark the odorants present in each trial (green for target, blue for nontarget). Some nontarget trials were repeated while others only appeared once. Trial types are pseudo-randomly interleaved.”

We also edited the schematic of Figure 1b-c to make the trial interleaving more obvious. We also hope that this change, along with re-numbering the trials, made the schematic more clear that the same mouse is licking left and right, being exposed to both Target and Nontarget odor mixtures in the same session. The new, edited panels are re-printed below:

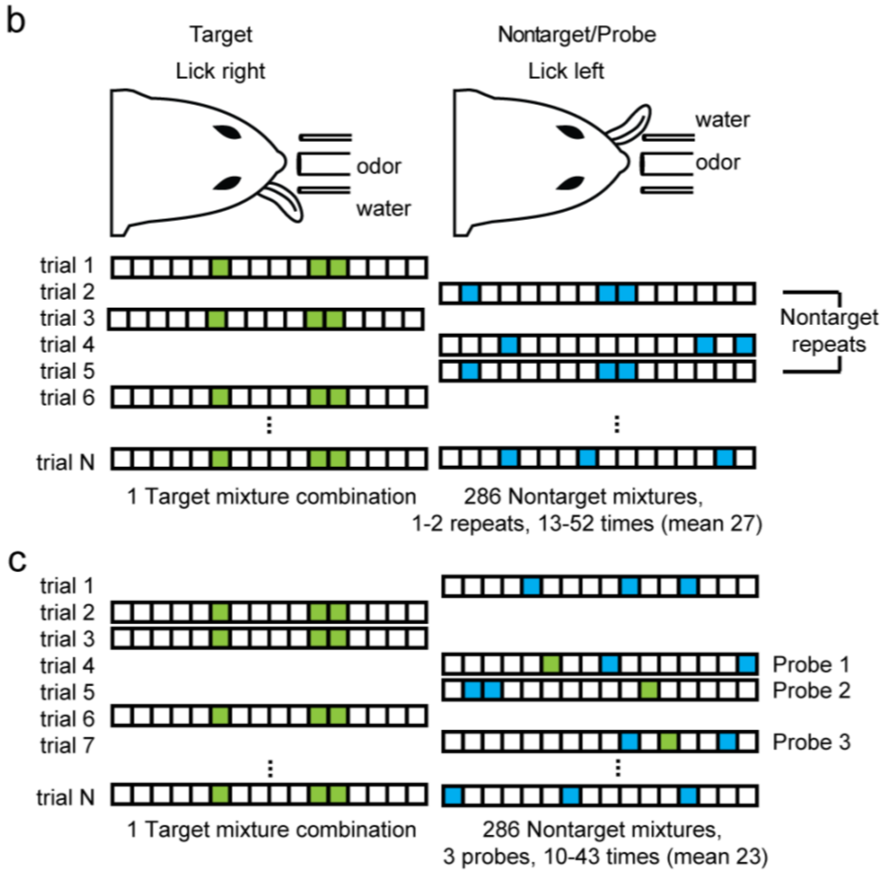

2) The data supporting improved category decoding with overtraining is difficult to evaluate. There is a small effect, supported by statistical analysis, with later sessions showing slightly improved decoding accuracy. However, day-by-day analysis reveals e.g. high accuracy in sessions 12 and 17, and low accuracy in sessions 23 and 24. Sessions 9 through 11 show consistently low accuracy and could still represent initial learning - would the statistical analysis still show a difference if sessions 12 through 28 were analyzed? Incorporating additional, more detailed behavioral measures (such as shown in Figure 5a, b) could possibly strengthen these analyses and support the authors' claim.

We thank the reviewer for their attention to the detail of the analysis presented in Figure 6c. We understand that the decoder accuracy is in flux, and not a steady rise across

sessions. However, we believe that we portray the data in a way that readers can see that this variability (which we would expect with the small sample sizes of the simultaneously recorded populations) exists. While it is true that you may expect different results by excluding sessions 8-11, you would also expect different results by excluding only 8-12 or only 25-28. We believe that cherry-picking the data to make claims of where the most change is happening would be inappropriate.

We also realized after reading this Reviewer as well as Reviewer 3's points, that we did not clearly communicate that Figure 6c is only including correct trials. This is clear from the Methods but was missing from the Results and Figure legend, so we have updated both of these sections with that information.

We have updated the figure legend to read:

"(c) The mean decoder accuracy across days during correct trials for each trial type."

We have also updated the Results to say:

"We found that the decoder performance during correct trials increases across days for target, nontarget, and nontarget repeat trials (Figure 6c; Table 1, row 17-19)."

In addition, the ANOVA for the data from Figure 6b is in Row 16 of Table 1 and shows that both session number as well as accuracy matter for the decoding performance.

3) The description of the analyses and figure panels for Figure 7 is confusing. Statements such as 'averaging the blue and green dots to get the pink dot' are not helpful. A more extensive depiction of the population vector correlation structure (such as in Figure 7c) would likely be more informative. Same trial population vector correlations of ~0.2 appear very low - please comment and clarify.

We thank the reviewer for pointing out that the main text lacks detailed explanation. We have edited the Results paragraph concerning these results to add an clarification of the population vector correlations, reprinted below:

"We used pairwise similarity analysis to assess how strongly the piriform population separates target and nontarget representations. The normalized Pearson's correlation coefficient of the simultaneously recorded neurons was taken for each trial (Figure 7c and top of Figure 7d; Supplementary Figure 7; see methods: Population vector correlations). For each target or nontarget trial, we calculated the 'target/nontarget similarity,' which is the average population vector correlation between that trial (e.g. a target trial), and all of the opposite (e.g. nontarget trials) category's population vectors (Figure 7d, each blue and green dot represents the 'target/nontarget similarity' for that trial and comes from averaging the blue and green lines). We predicted that the state of the target/nontarget representation during each probe trial could predict how the mouse would perform on that probe trial. To estimate the state of the cortex on each probe trial (pink dot in Figure 7d), we averaged the target/nontarget similarity in the ten trials preceding the probe trial and plotted this value for each probe trial (Figure 7d-e; averaging the blue and green dots to get the pink dot in d and in e, averaging the black dots to get the blue and red markers). This produced an extrapolated value of the

target/nontarget similarity during probe trials based off the running average of the last ten trials."

We also added clarification to the Figure 7c panel description in the figure legend, as such:

"(c) An example of population vector correlations (the Pearson's correlation of the activity of piriform neurons in two different trials) across trials in one session, averaged within trial-type categories (P1-3 are probes 1-3)."

The reviewer also noted that the population vector correlations appear low. We believe that this reflects the variability of the task encoding in the relatively low numbers of simultaneously recorded neurons. None of the correlations are done on pseudo-populations, where neurons are concatenated across days, but rather done using neuron populations recorded during any one session. This means activity in these piriform neurons that varies across trials will bring correlations down. This could include differences in sniff cycles, coding of other task variables, slight differences in the concentration, or various internal states.

4) What is 'overlearning'? I understand that the mice simply keep doing the task without improving their performance, which may impact odor coding and behavior. This is a potentially interesting point for further investigation, however, a more thorough introduction and discussion, for olfaction and more generally, could provide the necessary context.

We agree with the reviewer regarding the overlearning/overtraining that we see and describe in the study. We believe our discussion in the Importance of Training History section of the discussion does explain our thoughts of the most relevant literature, however we have also expanded this discussion to add more context. We describe another known effect of overlearning, the "overtraining reversal effect," in the added paragraph, re-printed below.

Training history has also been known to affect cognitive flexibility, reflected in the "overtraining reversal effect" (Sutherland & Mackintosh, 1971), whereby the overtraining of a task results in better performance during a subsequent change in the task. These studies predominantly concern spatial and visual tasks, but effects have also been seen in odor-guided tasks (Dhawan et al., 2019).

Minor points:

5) All relevant individual data points rather than the mean +/- SD only should be shown consistently, e.g. missing from Figures 1e-g.

We agree and we have adjusted Figure 1e-g to show individual data points.

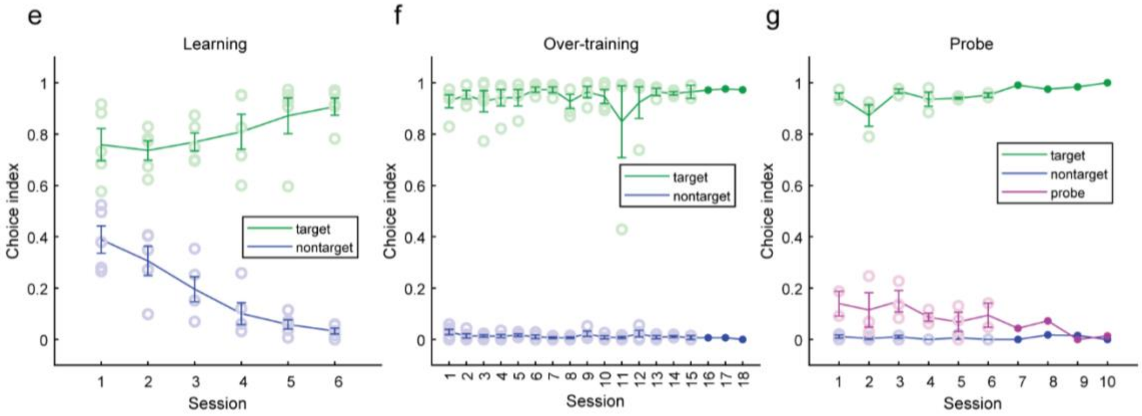

6) Language can be simplified throughout the description of the experiments, analyses, figure legends, and methods. The manuscript also contains a few typos and grammar mistakes that should be corrected.

We have corrected typos (also pointed out by Reviewer 1). We have also changed all instances of "overlearning" to "*overtraining*," for added clarity, as we realize we were using the two words interchangeably. We have made other adjustments to wording to make the language more clear as we addressed other concerns, but are open to any other suggestions of different, simplified, language.

Reviewer #3: Summary

Berners-Lee et al. examined population activity in posterior piriform cortex (PPC) in head-fixed mice performing a novel 2 alternative forced choice odor-mixture task. They first show that mice can learn this complex odor-mixture task, in which a target odor mixture needs to be differentiated from a series of different non-target mixtures. They then go on to demonstrate that the PPC neurons distinguish between target and non-target mixtures more than other comparative odor-mixture pairs (non-target, probe etc). The authors interpret these results as olfactory learning-dependent signals in PPC. They also show that during over-training, where behavioral performance has plateaued, PPC ensembles continue to increase their decoding performance between target and non-target odor mixtures. Overall, the experimental results are novel, substantial, and significant. In particular, I found the quantification on the diversity of individual neuronal responses in PPC thorough and interesting. The task design is also innovative and interesting. These results will have significant implications for piriform cortex function and the field of olfactory research in general. However, there are several major concerns I have with the data, analysis, and framing. In general, I found the framing and discussion a bit lacking in reference to the state of the field and recent advances. Additional experiments, analyses, and modifications in the text should be considered and incorporated in the revision.

Major Comments:

1. In the results presented here, we cannot parse whether target odor mixes are better decoded from PPC because 1) it is the stimulus most frequently presented to /experienced by mice; or 2) because it is the learned target stimulus. In other words, if untrained mice were passively exposed to the same sets of odor mixtures (target, non-target, probe etc.) with the same relative frequencies without having to perform the task, would the authors observe similar differences in decoding performance in PPC? Do the authors have access to this data?
2. Related to previous point, a recent paper (Schoonover et al. 2021 DOI: 10.1038/s41586-021-03628-7) showed that repeated passive exposures to odors stabilize and reinforce odor representations in the piriform cortex. As the authors stated in the first paragraph of the first section in their discussion: "The largest over-representation was of the target odor mixture, likely showing the effect of both the repeated exposure of the odor-mixture and the reinforce dimension of the task". If the authors cannot disentangle whether the effects they see are due to learning of the task, the title ("Learning-dependent evolution…") and emphasis of the entire paper should be rephrased/reworked. This is particularly critical given the finding reported here, where odor information present in PPC population is uncorrelated with task performance.

We thank Reviewer 3 for their careful assessment and insightful comments on our manuscript. We agree with the reviewer that because of the task design we are unable to separate experience from learning, although both are forces at play.

We do not, unfortunately, have access to the data the reviewer requests, however we have made changes to the main text and title in a way that we hope communicates the findings more clearly and precisely.

Schoonover et al. mainly focus on differences in the stability of representation (drift) with exposure (which we cannot evaluate, since we do not track the same neurons across days, but only take snapshots of the population activity each day), and not over-representation. There is, however, other evidence of over-representation with exposure. As suggested, we have added a paragraph to the discussion as explanation of the shortcomings and context of this study, re-printed here:

"Our data also does not speak to how much of the over-representation of target-selective neurons occurs from repeated experience or from learning. We do not have neural data from early learning to see how this over-representation emerges, and the target condition is the most frequent as well as the most reinforced. Both experience (Pashkovski et al., 2020) and reinforcement (Calu et al., 2007) have led to over-representation of selective neurons within the piriform cortex, and we believe both forces are at play here. Schoonover and colleagues also found that experience alone can also reduce drift of the aPC neural representation over time (Schoonover et al., 2021). Exploration of that effect cannot be assessed here, as we did not follow the same neurons over days. It is likely that the active component and the go-go structure of our task both contribute to differences in pPC representation along with the mere exposure of odors (Wang et al., 2019), and future work exploring how each of these task demands change separately across time would be fruitful."

As suggested, we have also changed the title of the paper to "*Experience-dependent*" from "learning-dependent" so as not to mislead the reader.

3. As stated in "Analysis of Behavior", trials in which mice responded before 500ms after odor onset were excluded from analysis. It is not justified why the authors chose this criterion. Please include trials in which responses were earlier than 500ms after odor onset. Specifically, 500ms is very long given what is known about the speed of olfactory decision making in rodents. See for example, Uchida and Mainen 2003 DOI: 10.1038/nn1142; Resulaj and Rinberg 2015 DOI: 10.1523/JNEUROSCI.4693-14.2015; Rinberg et al., 2006 DOI: 10.1016/j.neuron.2006.07.013). It is also not clear why mice lick with such long temporal delay as compared to previous reports. This is particularly misleading and problematic for analysis in Fig 5b.

We thank the reviewer for the opportunity to justify this 500ms criterion and have made changes to the main text and a supplemental figure to do so.

The vast majority of licks were recorded after this 500ms time. We have added a panel to Supplementary Figure 8 (panel A) which shows the full distribution of lick latencies (re-printed below).

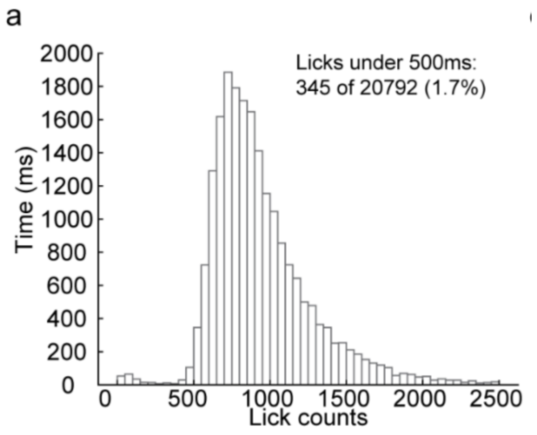

As it shows, less than two percent of the licks fall in this early range. It is, therefore, unsurprising that the results of analyses remain almost identical when these early licks are included. For example, the results of Figure 5b are shown before including the early licks:

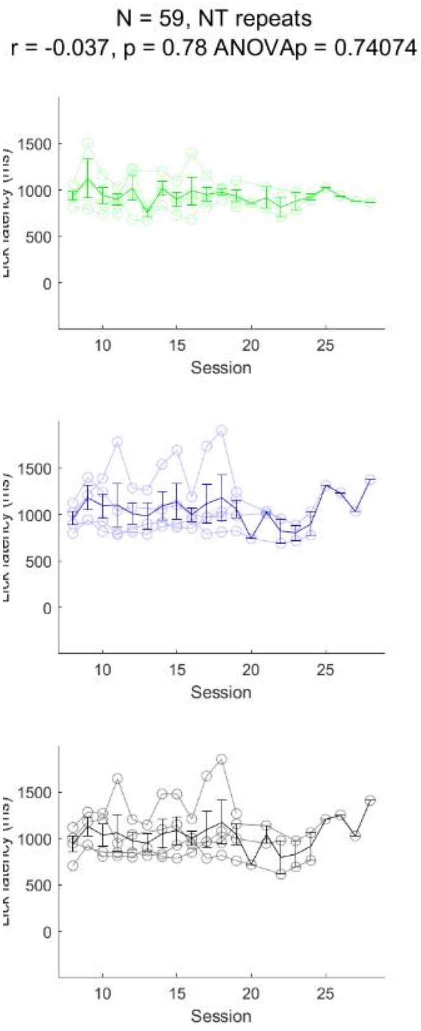

These results are qualitatively and quantitatively very similar, showing no effect of lick latency over time in any and all trial types.

We have also expanded on our description of the removal of these trials in the "Analysis of behavior" section of the Methods, re-printed here:

"We excluded the small number of trials (1.7%) in which the first lick came before 500 ms after odor onset (Supplementary Figure 8). Subsequent analyses, such as the lack of change in lick latency across overtraining, remain remarkably similar qualitatively and quantitatively when these short latency trials are instead included."

The reviewer also comments that it is not clear why mice lick with such long temporal delays. There are many aspects that are contributing to this longer delay than one may expect.

Firstly, a small part of these reaction times can be attributed to the delay of the odor reaching the nose, as well as the timing of the first sniff. As we state in the Analysis of Behavior section of the methods:

"Odor onset is demarcated as when the last solenoid switched on. There was a consistent delay between this time and when the odor reached the mouse which was about a hundred milliseconds."

Secondly, the difficulty of the olfactory task matters. The studies cited by the reviewer demand that the rodents either discriminate between two single odors in mixtures of different ratios (Uchida and Mainen, 2003; Rinberg et al., 2006) or between different concentrations of a single odor (Resulai and Rinberg, 2015). Even within these studies the reaction time often covaried with the difficulty (e.g., "to reach the same level of accuracy for harder tasks, the mouse needed to sample the odor for longer times [Rinberg et al., 2006; see also Figures 6-7].) Mice in the present study were exposed to many different odors in the same session and many more combinations of odors. In another study (Rebovich et al., 2021 DOI: 10.1371/journal.pcbi.1009674) where mice needed to discriminate one target odor within a background of other many other odors, reaction times were more similar to those in the present study (~500ms). Also, within that study, because of the difficulty of the task, lower reaction times were actually associated with incorrect choices.

Thirdly, the behavioral setup may be important for an animal's reaction time. The studies citied by the reviewer measured responses either by port entries in a freely-moving task (Uchida and Mainen, 2003; Rinberg et al., 2006) or by the direction of the mouses movement on a moving treadmill (Resulai and Rinberg, 2015). The present study's setup was head-fixed, where the decision was made by licking one of two lick ports. In studies with similar behavioral setups (including Rebovich et al., 2021, see also Bitzenhofer et al., 202,2 DOI: 10.7554/eLife.75065, Figure 2- figure supplement 1) reaction times are longer (~400-500+) and similar to ours. These longer reaction times are present even in non-olfactory tasks, like auditory (Marbach and Zador, 2017, DOI: 10.1101/073783) or visual (Burgess et al., 2017, DOI: 10.1016/j.celrep.2017.08.047) tasks, with this behavioral setup.

All these aspects that may be contributing to the observed reaction times in different degrees. We prefer to leave this discussion out of the paper since it does not affect the conclusions, but we are willing to add a few sentences highlighting the above points.

4. Fig 4a. It is unclear if the orange and yellow refer to modulated or selective neurons. In the figure legend, both words are used. Modulated would refer to a difference from 'baseline' (however that is defined) firing rate. Selective would refer to a test across two odor conditions (eg. between target and non-target). In this case, it is not clear which it is. If it is referring to target selective, and non-target selective neurons, what are the responses compared against?

We agree with the reviewer in their understanding of the meaning of modulated and selective. The bars in Figure 4a represent whether the neuron is modulated (up or down from baseline), whereas the colors and the key represent whether the neuron is selective (non-selective in blue, selective in yellow and orange).

Neurons labeled Target fire more to target than nontarget, neurons labeled Nontarget fire more to nontarget than target. We believe this breakdown was not clear in the figure legend so we have added two sentences explaining this. The beginning of this figure legend now reads as such:

"Proportion of all downwardly modulated neurons (left) and upwardly modulated neurons (right) that are significantly selective for target or nontarget odors. Target/nontarget label here refers to which condition the neuron fired more to. (Example nontarget and target neurons shown in Figure 3a and b, respectively.)"

5. Are all trial types shown in Fig 4c presented in all sessions? Do the same data (units) go into 'target', 'probe', and 'NT repeat' analyses here? These categories do not appear necessarily mutually exclusive to each other. Is this correct?

The reviewer is correct, and we thank them for their careful read of the manuscript. In order to make this clearer for other readers we have also included a description in the main text of the numbers of sessions and neurons per session within these analyses, which reads as such:

"Note that different numbers of sessions (Target: 80, Probe: 21, NT repeat: 59) and neurons per session (Target: range = 3-30, mean = 13.2, median = 13; Probe: range = 5-26, mean = 12.1, median = 9; NT repeat: range = 3-20, mean – 13.5, median = 13) are included in these analyses."

6. Fig 6b. What is the authors' interpretation of the fact that decoding performance is better for correct trial as compared to error trials, however there is no correlation between overall decoding and performance? I understood data from Fig. 6b to include 'overtrained' sessions. Is this correct? I would have expected no difference in decoding accuracy for correct vs. error trials for over-trained sessions.

Our interpretation of the fact that decoding performance is better for correct as compared to error trials is that the mouse is using the piriform cortical neurons' firing rates to help guide their decision-making. We do find a correlation between overall decoding and performance (same figure, Pearson's correlation). If outliers (mean-3std is

below ~0.56 mouse accuracy) are excluded, this correlation's significance vanishes. Our interpretation is that poor performance is related to low decoding, but at a steady-state high performance, decoding can continue improving with time. We had expressed this in the Results by saying:

"This is what we would expect if the mouse were using piriform cortex information to make its decision. We predicted that we would see an improvement in decoding across days, as the mice gain more experience, even though they were already at ceiling levels of performance."

In addition, the ANOVA the we performed to look at the data in figure 6b (found on Row 16 of Table 1) includes session as well as accuracy, and both are significant effects.

The data from Figure 6c and statistics in Rows 17-19 of Table 1 is also only from correct trials. Upon re-reading, we realized that this is clear from the Methods but not mentioned in the Results or the figure legend. We have updated the figure legend to read:

"(c) The mean decoder accuracy across days during correct trials for each trial type."

We have also updated the Results to say:

"We found that the decoder performance during correct trials increases across days for target, nontarget, and nontarget repeat trials (Figure 6c; Table 1, row 17-19)."

7. What does the PPC population activity look like during initial learning? Do the authors have access to this data? If there is no data on this, please include in the discussion a deeper consideration on what one would expect given results reported here.

Unfortunately, we do not have the early learning neural data. We agree with the reviewer that it would be interesting to see, and have added a paragraph to the discussion, as suggested, re-printed here:

"Our data also does not speak to how much of the over-representation of target-selective neurons occurs from repeated experience or from learning. We do not have neural data from early learning to see how this over-representation emerges, and the target condition is the most frequent as well as the most reinforced. Both experience (Pashkovski et al., 2020) and reinforcement (Calu et al., 2007) have led to over-representation of selective neurons within the piriform cortex, and we believe both forces are at play here. Schoonover and colleagues also found that experience alone can also reduce drift of the aPC neural representation over time (Schoonover et al., 2021). Exploration of that effect cannot be assessed here, as we did not follow the same neurons over days. It is likely that the active component and the go-go structure of our task both contribute to differences in pPC representation along with the mere exposure of odors (Wang et al., 2019), and future work exploring how each of these task demands change separately across time would be fruitful."

8. The authors do not sufficiently distinguish/discuss functional and anatomical differences

between anterior and posterior piriform cortex (APC vs. PPC). Throughout the manuscript, results in APC were cited interchangeably as evidence in support of the authors data and interpretation. While I recognize that there have not been very many studies published on PPC besides work from the authors' group. And that results in APC could serve to motivate experiments and guide framing for results in PPC, an explicit acknowledgement of the distinction between the two should be highlighted, and discussion on the potential functional distinction between the two regions should be expanded. Currently, this is only discussed in 2 sentences in the first paragraph of Introduction on page 3. I think the authors can do a better and more thorough job on this. There are several recent findings that suggest a functional and/or anatomical difference between APC/PPC (for example, Wang et al., 2020 DOI: 10.3389/fncir.2020.00004; Chen et al. 2022 DOI: 10.1016/j.cell.2022.09.038). The authors should cite and discuss such previous work, and discuss/frame their results in context of the field.

We have added a paragraph to the discussion about differences between anterior and posterior piriform, re-printed here:

"This study only considers pPC neurons, and there is reason to believe that aPC neurons could behave differently. There are anatomical and structural differences between anterior and posterior piriform (Chen et al., 2022; Hagiwara et al., 2012; L. Wang et al., 2020). These are accompanied by differences in encoding, with pPC being described as more associative and less sensory-driven than aPC (Calu et al., 2007; Haberly, 2001)."

We have also clarified whether anterior or posterior piriform was recorded from when discussing findings from other work. These changes are re-printed below:

Introduction:

"The piriform cortex, made up of the anterior piriform cortex (aPC) and posterior piriform cortex (pPC), is thought to be important in associative learning (Blazing & Franks, 2020; Gottfried, 2010; Haberly & Bower, 1989; Poo et al., 2022; Wilson & Sullivan, 2011). Indeed, when a single odor is paired with reward, although pPC neurons have not been found to encode odor valence (Gadziola et al., 2020; Millman & Murthy, 2020), more aPC neurons are often recruited to respond to those odors compared to unrewarded odors (Gire et al., 2013; Roesch et al., 2006). In the pPC specifically, more neurons are recruited to distinguish between two single odors that can lead to obtaining reward or avoiding punishment (Calu et al., 2007)."

Discussion:

"Perhaps this temporal refinement and truncation is due to recurrent circuitry within the pPC itself (Bolding et al., 2020; Bolding & Franks, 2018)."

"Odor-off responses have been reported in other species in olfactory receptor neurons and antennal lobe (Kurahashi et al., 1994; Nagel & Wilson, 2011; Nizampatnam et al., 2022), as well as in mouse aPC (Tantirigama et al., 2017)"

9. Related to previous point, there are additional gaps in citation and discussion on work in PPC which is directly relevant to the authors findings. This seems particularly unnecessary given the very few publications focused on PPC. For example: odor template matching: Zelano et al. 2011

DOI: 10.1016/j.neuron.2011.08.010, and associative learning: Poo et al. 2022 DOI: 10.1038/s41586-021-04242-3. In addition, while their results are in APC, Schoonover et al., 2021 DOI: 10.1038/s41586-021-03628-7 should also be cited and discussed in the context of the major observation of the paper (over-representation of target odor mixture). This is also relevant to the author's results in PPC decoding during overtraining.

We have added discussion of Schoonover and colleagues' work in a discussion paragraph re-printed below:

"Our data also does not speak to how much of the over-representation of target-selective neurons occurs from repeated experience or from learning. We do not have neural data from early learning to see how this over-representation emerges, and the target condition is the most frequent as well as the most reinforced. Both experience (Pashkovski et al., 2020) and reinforcement (Calu et al., 2007) have led to over-representation of selective neurons within the piriform cortex, and we believe both forces are at play here. Schoonover and colleagues also found that experience alone can also reduce drift of the aPC neural representation over time (Schoonover et al., 2021). Exploration of that effect cannot be assessed here, as we did not follow the same neurons over days. It is likely that the active component and the go-go structure of our task both contribute to differences in pPC representation along with the mere exposure of odors (Wang et al., 2019), and future work exploring how each of these task demands change separately across time would be fruitful."

We have also added Poo et al to the introduction as such:

"The piriform cortex is thought to be important in associative learning (Blazing & Franks, 2020; Gottfried, 2010; Haberly & Bower, 1989; Poo et al., 2022; Wilson & Sullivan, 2011)."

Minor Comments:

2. Page 4. 6th line from the bottom, the phrase "unlearned mixture" is confusing. "Unlearned" could be mis-interpreted to mean something that is first learned, and then unlearned afterwards. Consider rephrasing.

Changed to "irrelevant" mixture.
