# Supplementary material for: Experience-dependent evolution of odor mixture representations in piriform cortex
Source: PLoS Biol. 2023 Apr 25;21(4):e3002086. doi: 10.1371/journal.pbio.3002086 (PMC10129003; doi:10.1371/journal.pbio.3002086)
Supplement: Attachment — Submitted filename: Target_paper_responses_to_reviewers.pdf [file pbio.3002086.s011.pdf]

The previous submission (the second, revised one) was not sent out to the reviewers and only evaluated by the editor. Therefore, there are no reviewer comments to address, only editorial requests. We have been able to address all these requests (our responses in bold).

1) COMMENT FROM ACADEMIC EDITOR: As mentioned, the Academic Editor is largely satisfied by your response to reviewers and the changes made in this revision. However s/he has raised one lingering request. Regarding Reviewer 3's point 3 - the issue of the long latency between odor presentation and behavioral response - We think that you should add a few sentences on this topic in your manuscript, reflecting the points made in your response to reviewers.

We have added a section to the Discussion (page 23 of the revised manuscript, changes highlighted), where we highlighted the points outlined in our Response to reviewer 3 question 3. In making these changes, we added references 19-25.

2) ETHICS STATEMENT: Thank you for providing an ethics statement in your methods section indicating that all experiments were performed in accordance with the guidelines set by the National Institutes of Health and approved by the Institutional Animal Care and Use Committee at Harvard University. Can you please update this statement to include which specific national guidelines your protocol adhered to? We also request that you add the approval number for your animal care protocol approved by Harvard's IACUC.

We have updated the ethics statement (page 30), which now reads "Experiments were conducted in accordance with Harvard University Animal Care Guidelines and the "Guide for the Care and Use of Laboratory Animals" (National Research Council, 8th edition, 2011). Protocol 29-20-4 was approved by Harvard Faculty of Arts and Sciences' Institutional Animal Care and Use Committee".

3) BLURB: Please provide a blurb which (if accepted) will be included in our weekly and monthly Electronic Table of Contents, sent out to readers of PLOS Biology, and may be used to promote your article in social media. The blurb should be about 30-40 words long and is subject to editorial changes. It should, without exaggeration, entice people to read your manuscript. It should not be redundant with the title and should not contain acronyms or abbreviations.

We have added a blurb in the manuscript submission portal.
"The neural representation of important features of the environment can change as an animal learns. Here, Berners-Lee, Shtrahman and colleagues find that salient odor mixtures become over-represented and distinct in the olfactory cortex of mice, allowing a better and more robust categorization of these stimuli."

4) DATA AVAILABILITY: Thank you for uploading the raw underlying data for your manuscript to dryad. Can you please add a sentence to each figure legend (including supplemental) referencing this data? For example, to each figure legend you can add the sentence "the underlying data for this figure are available for download from https://datadryad.org/stash/share/bC3NdXWDllJZYrRtq60q0WDQYhjZZuLulv91dm9WcYU

We have added this text and the data availability link into all figure legends with data, including Supplementary figures.

5) DATA NOT SHOWN: Please note that per journal policy, we do not allow the mention of "data not shown", "personal communication", "manuscript in preparation" or other references to data that is not publicly available or contained within this manuscript. Please either remove mention of these data or provide figures presenting the results and the data underlying the figure(s). (I noticed one instance of data cited as 'not shown' on pg 38 (line 723))

We have edited the manuscript to delete where it said "not shown" because it was misleading. We were not describing different data, just explaining another analysis, and we have expanded upon that explanation to make it clear. There is no other occurrence of such phrases in the manuscript.
